# Two-step traction-assisted endoscopic submucosal dissection for a gastric neoplasm using a clip with a traction band and thread

**DOI:** 10.1055/a-2155-7172

**Published:** 2023-09-15

**Authors:** Kei Nishioka, Mitsuru Esaki, Tsutomu Iwasa, Yosuke Minoda, Noriko Shiga, Haruei Ogino, Eikichi Ihara

**Affiliations:** 1 Department of Gastroenterology, Saiseikai Futsukaichi Hospital, Fukuoka, Japan; 2 Department of Medicine and Bioregulatory Science, Graduate School of Medical Sciences, Kyushu University, Fukuoka, Japan; 3 Department of Gastroenterology, Harasanshin Hospital, Fukuoka, Japan; 4 Department of Gastroenterology and Metabolism, Graduate School of Medical Sciences, Kyushu University, Fukuoka, Japan

The traction technique is a common assistance method for gastric endoscopic submucosal dissection (ESD) 1 2 3 . We previously developed intralesional traction-assisted ESD (ILT-ESD) 4 5 . Although this provides a favorable clear view of the submucosal layer by achieving intralesional elevation using clips with a traction band, there is one problem to be solved. The traction force decreases because of the reduced area of lesion attachment as submucosal dissection progresses. To resolve this problem, we further developed a two-step traction-assisted ESD (TT-ESD), where intralesional traction is performed in the first half and clip-with-thread traction is applied in the latter half of the submucosal dissection ( Video 1 ).

**Video 1** Two-step traction-assisted endoscopic submucosal dissection is performed for a gastric neoplasm using a clip with a traction band and thread.


A gastric lesion with a 15-mm diameter was located at the greater curvature of the gastric body. TT-ESD was applied to the lesion. A clip with traction band and thread were prepared, with the thread tied to the traction band ( Fig. 1 ). After the circumferential mucosal incision around the lesion had been completed, the clip with the traction band and thread was placed at the proximal margin of the mucosal flap ( Fig. 2 a ). Subsequently, the second clip was placed at the distal margin of the lesion by hooking the traction band ( Fig. 2 b, c ). Intralesional traction was achieved by the elastic force of the band between the clips ( Fig. 2 d ). Submucosal dissection was conducted with a clear view of the submucosal layer in the first half of the procedure ( Fig. 2 e ). In the second part of the procedure, when the intralesional traction force decreased, conventional clip-with-thread traction was applied to generate an effective traction force on the lesion ( Fig. 2 f ). Submucosal dissection was therefore completed with a clear view of the submucosal layer throughout the procedure, and en bloc resection was achieved without any complications.

**Fig. 1:**
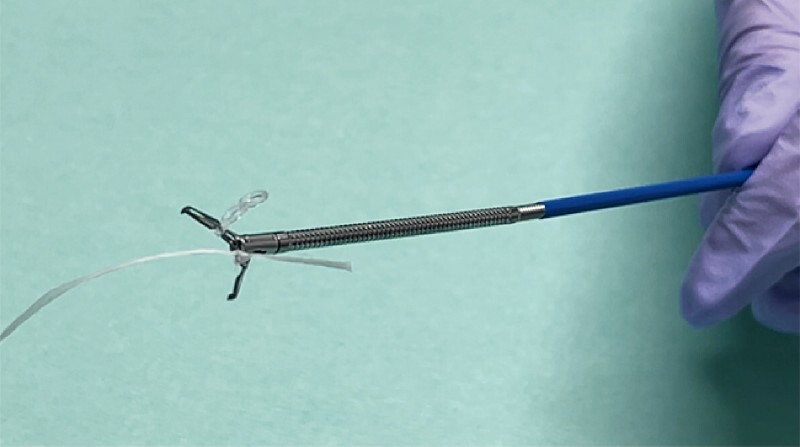
Photograph of the threaded clip that is created by manually tying floss onto the clip.

**Fig. 2:**
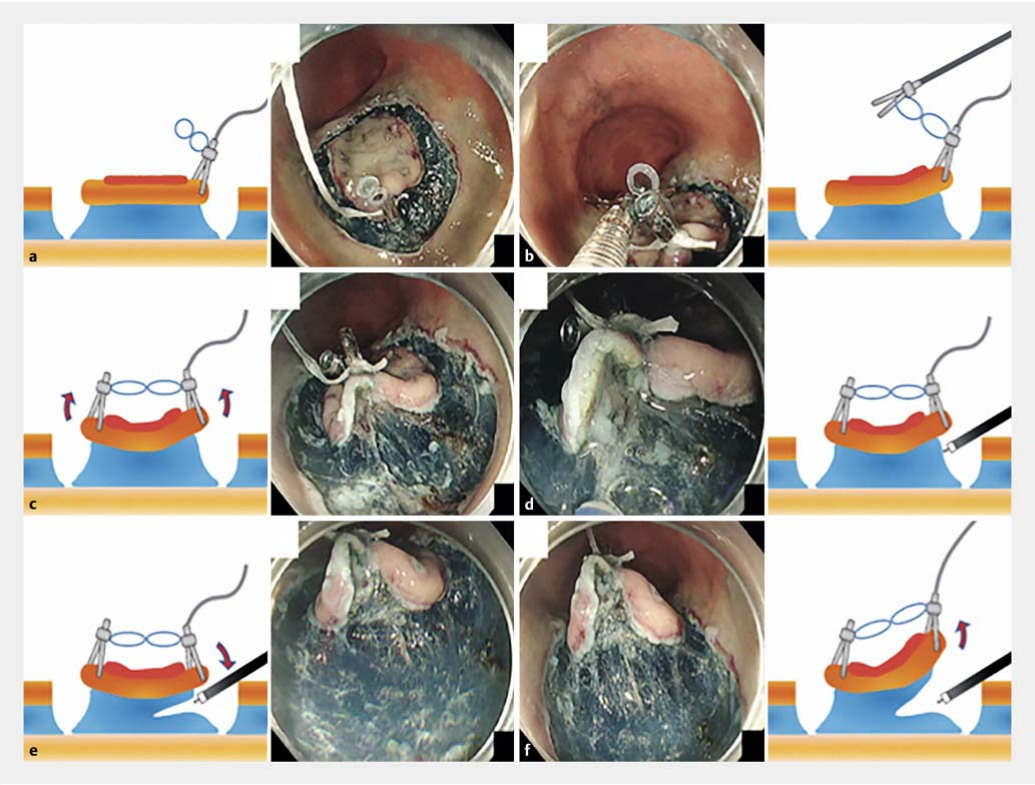
Endoscopic and schematic images of the procedure showing: **a** the clip, with the traction band and the thread tied to it, is placed at the proximal edge of the lesion; **b** the opposite ring of the traction band is hooked by the second clip; **c** the second clip is placed at the distal edge of the lesion; **d** intralesional traction is achieved; **e** submucosal dissection is started with a clear view of submucosal layer; **f** clip-with-thread traction is applied to the lesion once the effects of intralesional traction have decreased.

Intralesional traction assists the first half of the submucosal dissection, while clip-with-thread traction assists the latter stages of the procedure. This combined technique provides a clear view of the submucosal layer throughout the dissection procedure.

Endoscopy_UCTN_Code_TTT_1AQ_2AD

## Notes

Endoscopy E-Videos

https://eref.thieme.de/e-videos
*E-Videos* is an open access online section of the journal *Endoscopy* , reporting on interesting cases and new techniques in gastroenterological endoscopy. All papers include a high-quality video and are published with a Creative Commons CC-BY license. Endoscopy E-Videos qualify for HINARI discounts and waivers and eligibility is automatically checked during the submission process. We grant 100% waivers to articles whose corresponding authors are based in Group A countries and 50% waivers to those who are based in Group B countries as classified by Research4Life (see: https://www.research4life.org/access/eligibility/ ). This section has its own submission website at https://mc.manuscriptcentral.com/e-videos

## References

[1] Esaki M Ihara E Gotoda T Endoscopic instruments and techniques in endoscopic submucosal dissection for early gastric cancer Expert Rev Gastroenterol Hepatol 2021 15 1009 1020 33909540 10.1080/17474124.2021.1924056

[2] Yoshida M Takizawa K Suzuki S Conventional versus traction-assisted endoscopic submucosal dissection for gastric neoplasms: a multicenter, randomized controlled trial (with video) Gastrointest Endosc 2018 87 1231 1240 29233673 10.1016/j.gie.2017.11.031

[3] Suzuki S Gotoda T Kobayashi Y Usefulness of a traction method using dental floss and a hemoclip for gastric endoscopic submucosal dissection: a propensity score matching analysis (with videos) Gastrointest Endosc 2016 83 337 346 26320698 10.1016/j.gie.2015.07.014

[4] Shoguchi Y Esaki M Minoda Y Innovative endoscopic submucosal dissection for early gastric neoplasm using intralesional traction and snaring techniques Endoscopy 2022 54 02 E865 E866 35668660 10.1055/a-1841-5907 PMC9735343

[5] Shoguchi Y Esaki M Minoda Y Intralesional traction-assisted endoscopic submucosal dissection for early gastric neoplasm using the ProdiGI traction wire Dig Endosc 2022 34 e56 e57 35172386 10.1111/den.14239

